# Biodiversity and Health

**DOI:** 10.1093/eurpub/ckac129.021

**Published:** 2022-10-25

**Authors:** S Viegas

**Affiliations:** 1 NOVA National School of Public Health, Lisbon, Portugal; 2 Comprehensive Health Research Center, Lisbon, Portugal

## Abstract

Biodiversity underpins all life on Earth, and refers to biological variety in all its forms, from the genetic makeup of plants and animals to cultural diversity. Human health depends upon ecosystem products and services (e.g. availability of fresh water, food and fuel sources) which are requisite for human and animal health. Biodiversity loss can have significant direct human health impacts if ecosystem services are degraded and not able to guarantee social needs. The ecosystems also control disease and stabilize the climate. However, biodiversity loss is occurring at unprecedented rates, impacting human health worldwide, according to the report jointly published by the Convention on Biological Diversity (CBD) and the World Health Organization (WHO). In this report, awareness is provided to the need to promote integrated approaches to biodiversity and health by highlighting that biodiversity contributes to human health and wellbeing, and emphasizing also that biodiversity needs protection for development to be sustainable. Indirectly, changes in ecosystem services affect livelihoods, income, local migration and, on occasion, may even cause or exacerbate political conflict. Some features will be presented and discussed to describe in detail the most relevant impact that biodiversity has in human health and wellbeing and how the loss of biodiversity can imply risk for animal and human health.

